# Profiles of Whole Blood Transfusion Recipients at a Teaching Hospital in Lahore, Pakistan

**DOI:** 10.7759/cureus.21728

**Published:** 2022-01-30

**Authors:** Sana Haseeb Khan, Haseeb Ahmed Khan, Muhammad Ijaz Bhatti, Muhammad Mudasir Khan

**Affiliations:** 1 Pathology and Laboratory Medicine, Gulab Devi Hospital, Al-Aleem Medical College, Lahore, PAK; 2 Rheumatology, Gulab Devi Hospital, Al-Aleem Medical College, Lahore, PAK; 3 Cardiology, Gulab Devi Hospital, Al-Aleem Medical College, Lahore, PAK; 4 Internal Medicine, Jinnah Hospital, Lahore, PAK

**Keywords:** pakistan, hospital, pattern, utilization, blood transfusion

## Abstract

Background

Blood transfusion plays a vital role in medical practice. Evaluation of blood utilization in a blood bank is a crucial step in good transfusion practice. It is the inception towards assessing the present and future demands for blood and also avoiding unnecessary transfusions.

Methods

Retrospective analysis of one-year data, available in the blood bank of Gulab Devi Hospital Lahore, was done to study blood transfusion practice. Issue registers were accessed to retrieve the required information such as gender, age, blood group, pre-transfusion Hemoglobin level, ward, clinical diagnosis, and indication for the transfusion. The data was analyzed using SPSS version 20 software. Frequency and percentages were used to summarize categorical demographics and clinical variables.

Results

A total of 1181 units were requested from the hospital during our study period. Majority of the requests were for the female patients (69%) of the reproductive age group (21 to 30 years). Blood group B positive was most frequent in our study population. Maximum requests were from reproductive health departments (39.9%). Surgery was the major diagnostic category to require blood transfusions (31.8%). Elective surgery constituted the major indication requiring blood transfusion at our hospital (41.3%).

Conclusion

Blood utilization patterns vary significantly within geographical regions, hospital to hospital, and according to clinical practices as well as patients’ clinical findings. Moreover, diseases burden, level of organization, and advancement of healthcare facilities in various settings contribute to the significant contrast in blood utilization trends.

## Introduction

It is often quoted as a saying, "You can't manage what you can't measure." Unless you calculate something, you do not know if it is getting toward deterioration or improvement. You cannot estimate progress unless you measure what is improving and what is not. Blood transfusion performs a vital role in medical practice and is assumed to be the most common medical procedure undertaken by hospitalized patients [1]. To achieve good transfusion practice, continuous evaluation of the utilization of blood becomes essential [2]. This entails studying the pattern of blood consumption, clinical conditions and hospital wards requiring transfusion, and demographics of the blood transfusion recipients in a given population [3]. This evaluation is the key in assessing the present and future requirements for blood and also avoiding unnecessary transfusions.

The World Health Organization (WHO) reported a wide spectrum of demographics of the blood transfusion recipients according to their geographical regions. In developed countries, blood is most often utilized in trauma and cardiovascular surgeries, while in underdeveloped countries, obstetric conditions constitute the major burden on blood banks [4].

Transfusion safety includes three steps: reducing risk of pathogens’ transmission, appropriate transfusion thresholds, and adequate supply of blood. In our country, the basic focus confides only to the first step. Regular monitoring of demographic and clinical data of blood transfusion recipients of a country is very important as it is required to allow careful planning to prevent blood shortages or overproduction. To the best of our knowledge, this is the first-ever study from Pakistan of its type. It will assist in establishing local transfusion practice guidelines, streamlining scarce resources for the benefit of the patients, and conducting large-scale analysis in the future.

## Materials and methods

This study was a retrospective descriptive analysis carried out at the blood bank of Gulab Devi Hospital, Lahore, Pakistan. Gulab Devi Hospital has a capacity of 1,500 beds. Previously, it was a chest hospital (pulmonology and cardiology), but now for the past few years it is emerging as a multidisciplinary health care setup. Being a resource-limited institute, only whole blood is available in its blood bank.

The study was carried out over a period of one year, i.e., January 2019 to December 2019. Nonprobability convenience sampling method was used to conduct the study. Request forms of blood for all the patients who were admitted to the general wards of the hospital during the study period were analyzed. Issue registers of the blood bank were accessed to retrieve the required information such as gender, age, blood group, pretransfusion hemoglobin level, ward, clinical diagnosis, and indication for the transfusion. All the information was noted down manually in the research proforma by the researcher herself.

Wards of the hospital were divided into four major sections: medical, surgical, pediatrics, and gynecology/obstetrics. As mentioned earlier, Gulab Devi Hospital used to be a chest hospital, and request for blood from pulmonology and cardiology wards constitute a significant portion. Therefore, these wards were taken as separate sections from medicine. Diagnostic categories are derived from the International Classification of Diseases Version 10. Their primary and secondary codes were matched with our data, and the most frequent conditions requiring transfusion at our hospital were chosen.

The data were analyzed using SPSS Version 20 (IBM Corp., Armonk, NY). Frequency and percentages were used to summarize demographics and clinical variables. Gender, ABO and Rh blood groups, and diagnostic categories were expressed in tabulated forms, while age groups of transfusion recipients, their pre-transfusion hemoglobin level, ward, and indications of blood transfusion were represented using bar charts.

## Results

**Figure 1:**
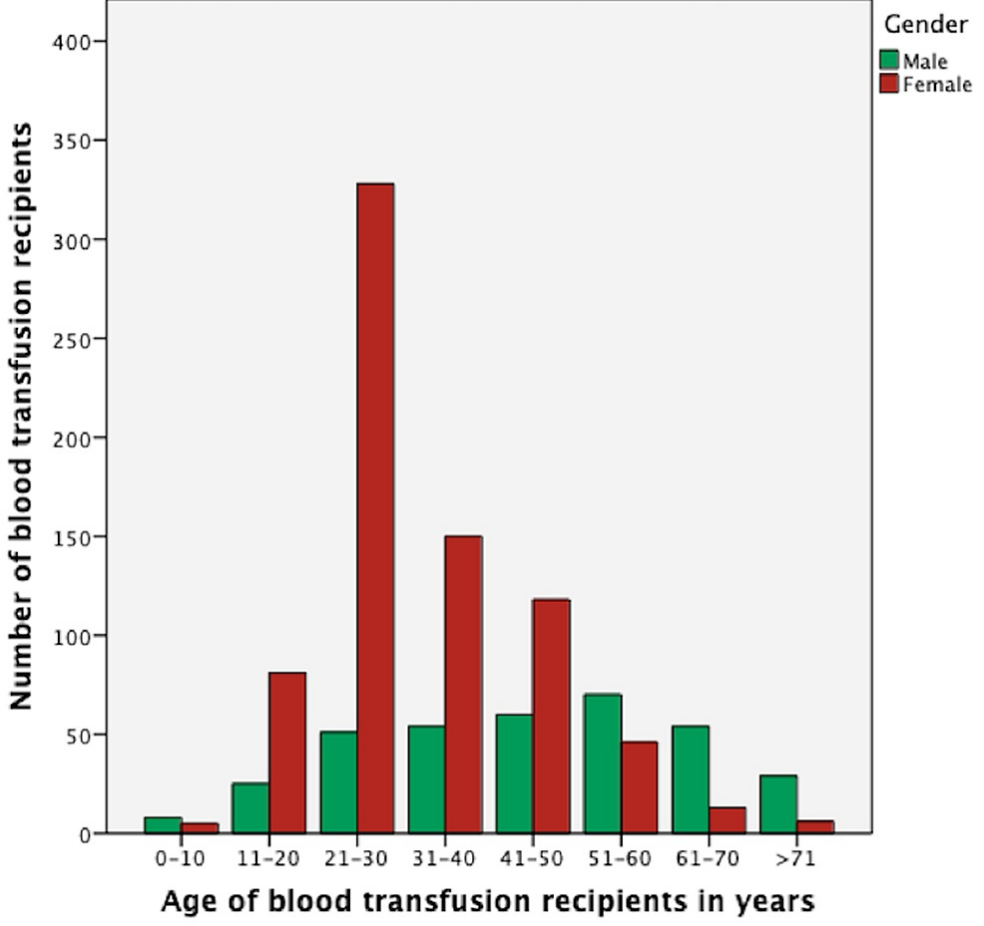
Age-wise distribution of transfusion recipients

**Figure 2:**
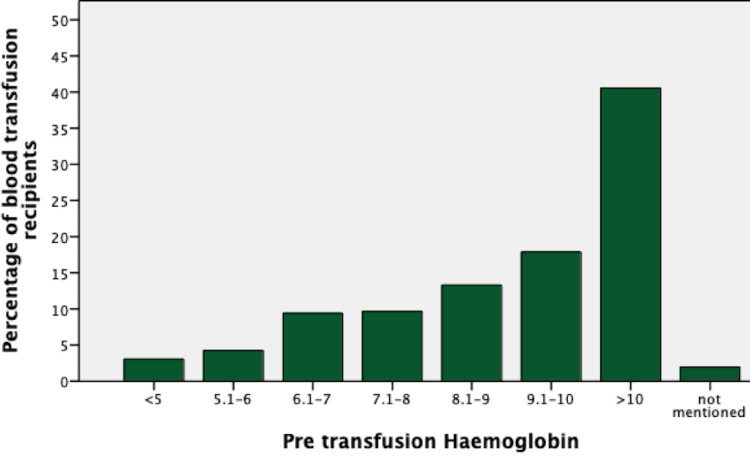
Pre-transfusion hemoglobin of recipients Units of hemoglobin are in grams/deciliter (g/dL)

A total of 1,181 whole blood units were requested from the hospital during our study period of one year, i.e., January 1, 2019, to December 31, 2019. Majority (69%; n=813) of the requests were for the female patients of the reproductive age group, while 31% (n=368) requests were for males (Table 1). The most frequent age group was 21 to 30 years, while the least frequent was 0 to 10 years (Figure 1). Majority of the patients (n=479; 40%) had a pre-transfusion hemoglobin level of >10g/dL (Figure 2). The most common blood group in recipients was B positive (n=381; 32.2%) while the least common was AB negative (n=5; 0.4%) (Table 2).

**Table 1:** Gender-wise distribution of transfusion recipients

	Males	Females	Total
Frequency (n)	368	813	1,181
Percentage (%)	31.2	68.8	100

**Table 2:** Frequency of ABO and Rh D blood groups in transfusion recipients + RhD positive; - RhD negative

	A+	B+	AB+	O +	A-	B-	AB-	O-
Frequency (n)	274	381	93	331	34	35	5	28
Percentage (%)	23.2	32.2	7.8	28.0	2.8	2.9	0.4	2.3

**Figure 3:**
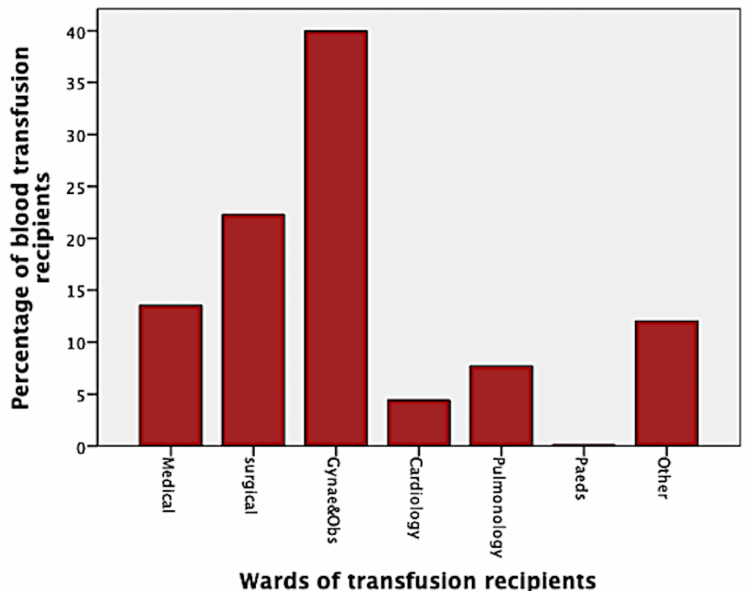
Distribution of blood transfusion recipients based on clinical wards Others include emergency; ear, nose, and throat; eye; intensive care unit; and psychiatry

Gynecology/obstetrics wards were at top with respect to blood requisition (n 471; 39.9%), followed by surgical wards (n=263; 22.3%) and medical wards (n=161; 13.6%). Pediatric units consumed the least number of blood bags (n=1; 0.1%) (Figure 3).

Surgery was the major diagnostic category to require blood transfusions (n=375; 31.8%), followed by pregnancy (n=362; 30.7%), diseases of blood and related disorders (n= 174; 14.7), puerperium and postpartum (n=82; 6.9%), pulmonary conditions (apart from tuberculosis) (n=79; 6.5%), caesarean section (n= 54; 4.6%); cardiovascular (IHD and CABG) (n=16; 1.4%), pulmonary tuberculosis (n= 15; 1.3%), cardiovascular (valvular disease/replacement) (n=9; 0.8%), neurological (n=8, 0.7%), neoplasms (n=4; 0.3%), and any other diagnosis apart from listed above (n=3; 0.3%) (Table 3).

**Table 3:** Distribution of blood transfusion recipients based on the broad diagnosis categories IHD, ischemic heart disease; CABG, coronary artery bypass grafting

Provisional diagnosis	Frequency	Percentage
Pregnancy	362	30.7
Puerperium and postpartum	82	6.9
Caesarean section	54	4.6
Pulmonary tuberculosis	15	1.3
Pulmonary (other disorders)	79	6.7
Neoplasms	4	0.3
Surgery (due to nonneoplastic reasons)	375	31.8
Cardiovascular: IHD and CABG	16	1.4
Cardiovascular: valvular disease/replacement	9	0.8
Diseases of blood and related disorders	174	14.7
Neurological	8	0.7
Others	3	0.3
Total	1,181	100.0

**Figure 4:**
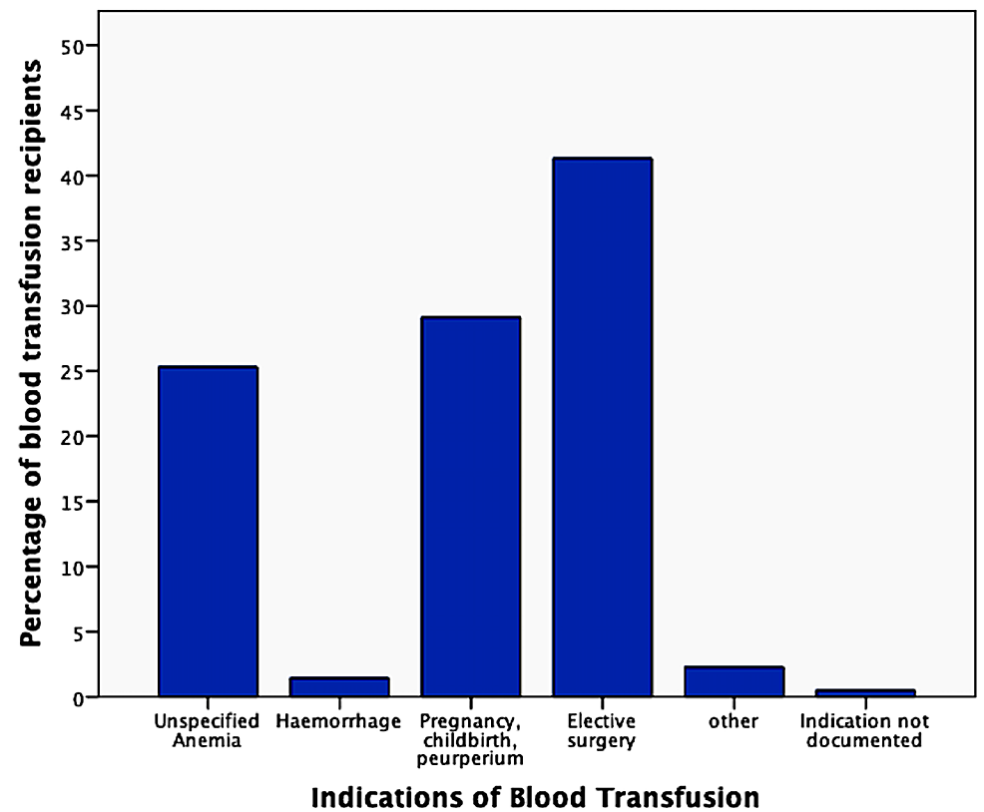
Indications for blood transfusion

Indication referred to the exact trigger or cause that leads to demand of transfusion at the particular point. We divided the indications of blood transfusions into four major categories. Elective surgery constituted the major indication requiring blood transfusion at our hospital (n=488; 41.3%). Other major indications for transfusions were pregnancy, childbirth and puerperium (n=344; 29.1%), unspecified anemia (n=299; 25.3%), hemorrhage (n=17; 1.4%), and any other indication apart from these four (n=27; 2.3%). There were few requests without documentation of any indication on request forms (n=6; 0.5%) (Figure 4).

## Discussion

Blood transfusion should provide a therapeutic advantage to the patients. Good transfusion practices should regularly monitor the utilization of the blood and its components to ensure adequate safety. This study provided data on the pattern of whole blood utilization, as well as demographic and clinical characteristics of transfusion recipients at Gulab Devi Hospital, Lahore, Pakistan.

Females of the reproductive age group were predominant transfusion recipients at our hospital (68.8%). This observation is consistent with some previous studies [3,5-7]. However, some of the previous literature reported males requiring more blood transfusion [8,9]. Female predominance may be explained on obstetric/gynecological grounds, as the patients from reproductive health section comprised a major portion requiring blood in our country.

Our study comprised a younger cohort of transfusion recipients. The most common age group with respect to utilization of blood was 21 to 30 years and the least frequent was 0 to 10 years. This finding is consistent with the previous literature from developing countries [2,3,5-7,9,10]. Our age spectrum differs from that of developed countries where most of the blood transfusions are utilized by the older age group [8,11,12]. This can be attributed to the median age of a certain geographical zone. As in developed countries such as Japan, Germany, and Italy, the median age of the population is around 48 years. Contrary to this, the median age in our country is found to be 22 years [13]. A report by WHO on blood safety and availability reported a vast diversity between various countries in terms of the age distribution of blood transfusion recipients. In high-income countries, the most frequently transfused age group is over 60 years, accounting for up to 75% of all transfusions. In low-income countries, around 54% of transfusions are received by children under five years of age [4].

The distribution of ABO blood grouping among the blood transfusion recipients at our hospital corresponds with that of the general Pakistani population [14,15]. It is pertinent for any blood bank to have knowledge of ABO blood group distribution among its recipients. Awareness of both institution-based and national ABO and Rh blood group patterns helps the blood bank to maintain a sufficient blood supply and rationalize its utilization.

The majority of the patients at the time of blood requisition had a hemoglobin of greater than 10g/dL, and most of the transfusions were designated to those requiring elective surgeries. Studies have reported lack of any extra benefit in cases associated with the use of “liberal” versus “restrictive” transfusion strategies [16]. This calls for formulating local hospital policies for transfusion triggers. Moreover, educating the surgeons/clinicians with updated international transfusion guidelines for optimal blood management is also of paramount importance. The hospital transfusion committee may review the reasons for administrating blood on this “high” hemoglobin level followed by a discussion with surgeons whose blood transfusion practices are consistently outside guidelines. This discussion and awareness should be followed by a further period of data collection on transfusion patterns in the hospital and further review as an iterative exercise of quality improvement.

Reproductive health departments (gynecology/obstetrics) followed by surgical units received the maximum number of blood bags at our center. Patients of medical wards consumed less blood overall as compared to surgical domains. This finding is akin to the previous studies from various countries [ 3,5,6,9,17]. In cases requiring surgeries, blood is often ordered due to anticipated rather than actual blood loss. Evidence of inappropriate transfusions in surgical and reproductive health departments is also reported in other countries [18,19]. In gynecology/obstetric, it should be reinforced that no woman enters the intrapartum period with preventable anemia to avoid such “over” transfusion practices.

The top diagnostic categories found to be demanding blood at our hospital were surgery (31.8%) and pregnancy (30.7%), followed by diseases of the blood and related disorders (14.7%). These results are related to some extent with that of previous studies from other countries. A study from India reported surgical cases to be mostly utilizing the blood followed by neoplasms [9]. Another study from Kenya reported neoplasms followed by pregnancy to be the major burden on transfusion demand [5]. Literature from Nigeria revealed conditions originating from childbirth and pregnancy to be top of the list for blood utilization [3]. Another study from Spain reported neoplasms, followed by endocrine, metabolic disturbances, and diseases of the blood and blood-forming organs to be associated with maximum transfusion demand [8]. Overall, in developed countries, blood utilization trends vary from that of the underdeveloped ones. A large-scale survey from developed countries also reported neoplasms to be the top diagnostic category requiring blood transfusion [20]. This variation is also supported by WHO that in high-income countries, transfusions are most commonly used for cardiovascular surgeries, massive trauma, transplants, and solid organ and hematological malignancies, while in low- and middle-income countries it is reserved for pregnancy-related conditions and childhood anemia [4]. There is a hospital-to-hospital variation in blood consumption depending upon the disease burden. In our hospital, we lack an oncology setup; therefore, neoplasms constitute a very small number of cases needing blood as compared to other countries. Moreover, Gulab Devi Hospital used to be a chest (pulmonology and cardiology) hospital previously. It has grown as a comprehensive health care setup for only a past few years. Therefore, cardiac and pulmonary diseases still manifest as prominent categories requiring blood.

Surgeons have a perception that all surgical cases would need blood at some point of their hospital stay. This liberal transfusion practice also leads to unnecessary exposure of the recipient to he blood-borne infections as well as foreign antigens. At times, the really needy patients may get deprived of the blood due to limited units. Strict adherence to surgical blood order schedules can improve this attitude. A study by Mauka et al. reported admission in the surgical wards as one of the independent risk factors for inappropriate blood utilization [21]. Perioperative surgical conditions have been linked with over-issuing of blood bags, consequently leading to its wastage. This study also claims that, overall, surgical patients are more at risk to be inappropriately transfused as compared to medical patients, and this has been associated with high morbidity and mortality. To reduce blood consumption in surgical domains, we need bloodless surgeries, but these advanced infrastructures have restrictive availability in developing countries.

Indication of the blood transfusion refers to the exact trigger that calls for transfusion at that moment. Elective surgeries, conditions relating to childbirth, and unspecified anemia were found to be the most common indications requiring transfusion at our hospital. Results of the previous studies on blood utilization yield variable results in this regard. Anemia is reported as the most common cause in some studies [2,5], while hemorrhage is the major indication in others [8,10]. Underdeveloped countries do not have facilities for bloodless surgeries. This leads to “over” transfusion at higher than indicated hemoglobin levels in surgical patients. Moreover, expected than actual blood loss in surgical domains is a quotidian practice in underdeveloped countries. Pre-operative anemia is also a frequent finding in reproductive health and surgical patients. Its incidence in surgical patients is as high as 75%, depending on the patient’s comorbidity, age, gender, and underlying pathophysiology requiring surgery [22]. Peri-operative anemia is higher in women than men, and women are at higher risk of bleeding during surgeries than men due to low circulating red cell mass. Peri-operative anemia is associated with prolonged hospital stay, slow recovery, readmission, and increased postoperative mortality and morbidity. A low pre-operative hemoglobin level (<13g/dL) is one of the most important predictors of blood transfusion, which, in turn, is another risk factor for poor outcomes in surgical cases [22]. Most of the blood utilization at our setup is from surgical and gynecology/obstetrics units, with anemia being an important underlying indication. Anemia and hematinic deficiencies in surgical and pregnant patients should be identified in a timely manner and classified and treated before any major procedure. For elective surgeries, this may entail postponing the procedure until anemia is improved or resolved. Despite the best efforts and organization, transfusions will still occur in surgical and obstetric cases, but preparations may reduce its burden.

Our study has several potential limitations. It has a relatively small sample size. It was carried out in a major health care institution in Lahore, Pakistan, but our hospital lacks a separate oncology department as well as a very limited pediatric setup. Pulmonology and cardiology wards face highest influx of patients at our hospital. Moreover, blood components are not being produced in its blood bank due to limited resources. For these reasons, extrapolation of overall findings of this study to the country may need caution. Moreover, in this study, we assumed that all the issued blood was transfused.

## Conclusions

Blood utilization patterns vary significantly within geographical regions, hospital to hospital, and according to clinical practices as well as patients’ clinical profiles. Moreover, level of organization, advancement of healthcare facilities, and diseases burden in the various settings contribute to the significant contrast in blood utilization. Although a single-center data, our study contributes insight into the demographic and clinical characteristics of blood transfusion recipients. It will provide the basis for planning more comprehensive studies on the utilization of blood as well as stimulate substantial interest in both hospital-based and national blood management initiatives to rationalize blood utilization and ameliorate patient outcomes. By a holistic approach involving the clinicians, surgeons, anesthesiologists, and blood bank staff, we can reduce the number and change the pattern of blood consumption, making it as appropriate as we can.
